# Between Aesthetics and Health: Disordered Eating, Exercise Addiction, and Body Image in Competitive Bodybuilders

**DOI:** 10.3390/jfmk11020236

**Published:** 2026-06-13

**Authors:** Federica Moro, Irene Cruccolini, Mario Mauro, Natascia Rinaldo, Emanuela Gualdi-Russo, Luciana Zaccagni, Stefania Toselli

**Affiliations:** 1 Department for Life Quality Studies, University of Bologna, 47921 Rimini, Italy; federica.moro10@unibo.it (F.M.); irene.cruccolini@studio.unibo.it (I.C.); mario.mauro4@unibo.it (M.M.); stefania.toselli@unibo.it (S.T.); 2 Department of Neuroscience and Rehabilitation, University of Ferrara, 44121 Ferrara, Italy; emanuela.gualdi@unife.it

**Keywords:** bodybuilding, disordered eating, orthorexia nervosa, exercise addiction, body image, RED-S

## Abstract

**Objectives**: To examine disordered eating behaviors, orthorexic tendencies, binge-eating episodes, attitudes toward exercise, perceived hormone-related symptoms and body image perception among competitive bodybuilders across different levels of competitive experience. **Methods**: In this cross-sectional study, 60 competitive bodybuilders (29 men, 31 women) completed an anonymous online questionnaire. The survey evaluated demographic characteristics, coaching and training management, phase-specific symptoms (such as libido, sleep, eating behaviors, and menstrual alterations), orthorexic tendencies, exercise addiction, and body-image perception. **Results:** Both sexes reported reduced libido, increased hunger, and sleep disturbances, along with frequent weight monitoring and common binge-eating episodes. Moreover, females frequently reported menstrual irregularities. ORTO-15 scores indicated a potential risk of orthorexia nervosa, while EAI-3 scores suggested a risk of exercise addiction in novice females and advanced males, with differences in mood regulation and guilt across sex and experience. Males showed higher perceived and ideal muscle mass, whereas females reported higher perceived body fat and a preference for leaner physiques. **Conclusions**: Competitive bodybuilders of both sexes exhibit post-competition binge eating, mood- and appearance-driven exercise behaviors, and pronounced body-image concerns. Screening, education on energy availability, structured post-competition support, and health-focused coaching are recommended to prevent the progression from sport-specific practices to clinical pathology.

## 1. Introduction

Bodybuilding is an aesthetic strength sport in which athletes are evaluated primarily on muscular size, definition, symmetry, and overall stage presentation rather than on physical performance outcomes [1,2]. To optimize these qualities, competitive bodybuilders typically structure their preparation around training and nutrition, which are organized into distinct phases. The off-season, or bulk, is characterized by a sustained energy surplus to maximize muscle hypertrophy, whereas the contest-preparation phase involves progressive caloric restriction to reduce body fat, while preserving lean mass. This is followed by a brief “peak week” immediately before competition, during which carbohydrate, fluid, and electrolyte intake are often aggressively manipulated to optimize muscular fullness, definition, and overall stage appearance [1,2,3,4].

Although these strategies can optimize physique, they also entail prolonged periods of low energy availability and repeated cycles of weight gain and loss, which may have negative consequences for endocrine function, physical health, and psychological well-being [4,5,6,7,8]. Recent research in physique athletes has shown that contest preparation may be associated with alterations in hormones regulating appetite, stress response, reproductive function, and energy balance, including changes in ghrelin, leptin, cortisol, and, in men, testosterone levels. These endocrine adaptations have been associated with symptoms such as increased hunger, reduced satiety, heightened stress, sleep disturbances, decreased libido, and menstrual cycle irregularities [7,8,9,10]. Collectively, these patterns are consistent with a condition of low energy availability and Relative Energy Deficiency in Sport (RED-S), which may impair reproductive, skeletal, metabolic, and immune function in both women and men [5,6]. Given prolonged caloric restriction and extremely low body fat levels, this sport may entail a particularly high risk of RED-S and related endocrine disturbances, especially in female athletes [10,11,12].

Within this physiological context, bodybuilding is also characterized by a strong emphasis on body image and appearance control. Body image disturbances therefore represent a central risk factor in bodybuilding. Body image is a multidimensional construct involving perceptual, cognitive, affective, and behavioral components through which individuals experience and evaluate their own bodies [13,14]. In aesthetic and strength sports, body dissatisfaction may focus on both adiposity and muscularity, with male athletes typically striving for greater muscle size and lower body fat, while female athletes often experience pressure toward both muscularity and thinness [15,16,17].

This may contribute to the development of eating disorders, defined in the Diagnostic and Statistical Manual of Mental Disorders (DSM-5) as persistent disturbances of eating or eating-related behaviors that significantly impair physical health or psychosocial functioning, and include anorexia nervosa, bulimia nervosa, and binge-eating disorder, among other specified and unspecified feeding and eating disorders [18]. In bodybuilding many behaviors that are considered normal or even necessary for competitive success, such as meticulous tracking of food intake, avoiding certain “off-plan” foods during contest preparation, and structuring the year around bulking and cutting phases, closely resemble behavioral patterns observed in eating disorders, thereby complicating the distinction between sport-specific demands and pathological eating and potentially exposing athletes at elevated risk [15,19,20,21]. Studies in physique and fitness athletes have reported elevated dietary restraint, increased cognitive preoccupation with food, post-competition increases in body shape concern, and a greater incidence of binge-eating episodes, particularly in the weeks following competition when caloric intake is liberalized while body image remains highly salient [11,22].

Orthorexia nervosa, a condition characterized by an obsessive focus on so-called “healthy” or “clean” eating and the rigid avoidance of foods perceived as impure or unhealthy, may be particularly relevant in bodybuilding, where nutrition plays a central role in performance and appearance [23,24]. Excessive preoccupation with food quality, strict dietary rules, and the dichotomisation of foods into “allowed” and “forbidden” categories can lead to nutritional inadequacies, social impairment, and significant distress, even in the absence of underweight or classic binge-purge behaviors [23,24]. Cross-sectional evidence suggests that bodybuilders report higher levels of orthorexic tendencies than non-athlete controls or other strength athletes, potentially reflecting the gradual crystallization of sport-driven dietary strategies into enduring, rigid patterns [25].

Exercise addiction or compulsive exercise also plays a central and often dominating role in an athlete’s life. This condition is marked by loss of control over exercise, withdrawal-like symptoms when unable to train, persistence despite injury or adverse consequences, and conflicts with work, social, or family life [26,27,28]. High-intensity resistance-training sports, including bodybuilding, are associated with particularly elevated scores on exercise-addiction measures, and excessive training may interact with low energy availability and disordered eating to exacerbate both physical and psychological risk [20,27,28].

Closely related to exercise addiction or compulsive exercise is body dysmorphic disorder, defined by a preoccupation with perceived defects in appearance and associated repetitive behaviors, or its muscle-focused subtype, defined as muscle dysmorphia, characterized by the belief that one is insufficiently muscular despite having a highly developed physique [13,29]. Research among male bodybuilders has reported high rates of muscle-dysmorphia symptoms, marked dissatisfaction with muscularity, rigid dietary practices, and frequent use of supplements or anabolic agents to increase muscle mass [21,30,31,32].

Personality traits, such as perfectionism and low self-esteem, frequently reported among physique athletes, may further amplify vulnerability to both body image–related psychopathology and disordered eating [15,32]. Importantly, recent evidence has highlighted a significant association between body image and exercise dependence, suggesting that body image concerns may contribute to maladaptive exercise behaviors, including compulsive exercise and exercise addiction [33,34]. Body image disturbances, disordered eating, and exercise addiction may therefore represent interconnected components of a broader maladaptive behavioral spectrum in aesthetic sports.

Despite this constellation of risk factors, the existing bodybuilding literature remains limited in several respects. Many studies have focused exclusively on female physique athletes [11,19,22] or exclusively on male bodybuilders [25,31,32], precluding direct comparisons between sexes. Moreover, competitive experience, an element likely to shape both exposure to extreme weight-management practices and internalization of sport-specific appearance ideals, has rarely been examined as a moderator of risk, and endocrine symptoms are often assessed indirectly or not at all [7,8].

The present study aims to address these gaps by assessing eating behaviors, orthorexic tendencies, binge-eating episodes, exercise-related attitudes, perceived hormone-related symptoms (e.g., hunger, satiety, libido, menstrual changes, stress and sleep), and body image self-perception in competitive male and female bodybuilders with different levels of competitive experience. We hypothesized that disordered eating behaviors, orthorexic tendencies, exercise-addiction risk, hormone-related symptoms, and body-image concerns would differ according to sex and competitive experience. By explicitly comparing sexes and experience levels within the same sport, this study seeks to clarify whether and how the occurrence and expression of disordered eating, orthorexia, muscle dysmorphia, and exercise addiction differ between male and female bodybuilders, and to provide a basis for targeted prevention and multidisciplinary management strategies aimed at safeguarding the physical and psychological health of this high-risk athletic population.

## 2. Materials and Methods

### 2.1. Study Design and Participants

This cross-sectional study involved 60 competitive bodybuilders recruited through multiple channels, including personal contacts, bodybuilding coaches who promoted the questionnaire to their athletes, and direct recruitment at bodybuilding competitions and competitive events. Inclusion criteria were: (i) age ≥ 18 years; (ii) active involvement in competitive bodybuilding; (iii) membership in a natural bodybuilding organization (AINBB or NBFI); and (iv) participation in at least one bodybuilding competition in the previous 12 months, or active preparation for a competition at the time of data collection. Exclusion criteria were the following: (i) recreational bodybuilders not involved in competitive bodybuilding; (ii) individuals not affiliated with a natural bodybuilding organization; and (iii) former competitors who were no longer actively involved in competitive bodybuilding.

The sample included 29 men and 31 women, who competed in the Men’s Physique or Classic Physique and in the Bikini or Wellness categories, respectively. Participants were stratified into three groups according to years of competitive experience, as follows:

• <3 years, defined as “novice”
• 3–5 years, defined as “intermediate”
• >5 years, defined as “advanced”

As no standardized classification of competitive experience is currently available for natural bodybuilders, these cut-offs were established a priori by the authors for analytical purposes. Participation was voluntary and anonymous. All athletes were informed about the study’s objectives and provided informed consent. The study was approved by the Bioethics Committee (Approval Code: 25027).

Data were collected via an anonymous online survey (Google Forms) from June 2025 to October 2025. Athletes completed the questionnaire independently; no identifying information was collected.

### 2.2. The Questionnaire

The online questionnaire consisted of five sections as follows:

#### 2.2.1. Background, Sport-Related and Anthropometric Characteristics

The first section collected sociodemographic and sport-related data, as well as anthropometric data. This included information on sex, age, years of bodybuilding experience, competitive experience category, height, current weight, and the minimum body weight achieved in relation to the competition (i.e., competition-day body weight). Additional questions assessed whether athletes were followed by a coach, whether training and dietary plans were prescribed by the same or different professionals, and whether a multidisciplinary team was involved.

#### 2.2.2. Preparation and Hormonal/Phase-Related Symptoms

The second section assessed characteristics of competition preparation and subjective symptoms related to hormonal status. In particular, all athletes were asked about decreased libido and sleep disturbances during preparation, assessed using binary (yes/no) response options. Moreover, ratings of perceived stress, irritability, hunger, satiety, and insomnia were collected using 5-point Likert scales across three phases: mass-gain, contest preparation, and post-competition. Additionally, for female athletes, the questionnaire evaluated menstrual cycle alterations, such as irregular cycles, amenorrhea associated with contest preparation, and their duration. All these measures were considered proxies of RED-S-related and preparation-related strain [5,6,7]. Information on specific competitive phase, training frequency, and supplement use was not collected.

#### 2.2.3. Eating Behaviour

The third section focused on eating-related behaviours, including frequency of body-weight monitoring (e.g., less than once per week, weekly, several times per week, daily, more than once per day), perceived impact of weight on mood (yes/no and 1–10 rating if yes), the presence and timing of binge-eating episodes across the bulk, preparation, and post-competition phases, and the use of extreme weight-loss strategies, such as diuretics, laxatives, fasting, self-induced vomiting, and compensatory exercise beyond planned training.

Furthermore, orthorexic tendencies were assessed using the ORTO-15 questionnaire [23], which assesses obsessive thoughts and behaviours related to healthy eating. The questionnaire consists of 15 items rated on a 4-point Likert scale ranging from “never” to “always”, with item-specific scoring procedures producing total scores between 15 and 60. Scores below 40 indicate a significant tendency toward orthorexia [23,32].

#### 2.2.4. Training and Exercise Addiction

The fourth section assessed training-related attitudes using the Expanded Exercise Addiction Inventory (EAI-3) [27]. This 8-item tool extends the original Exercise Addiction Inventory [35] by including 2 items on additional pathological features, such as guilt and training despite injury. Items are rated on a 6-point Likert scale from 1 (strongly disagree) to 6 (strongly agree), covering the following dimensions: salience, mood modification, tolerance, withdrawal, conflict, relapse, guilt, and training despite injury. A score equal to or higher than 34 can suggest a greater risk of exercise addiction [27].

#### 2.2.5. Body-Image Perception

The fifth section evaluated body-image perception and desired physique using sex-specific Body Image Matrices of Thinness and Muscularity. Specifically, men completed the Body Image Matrix of Thinness and Muscularity—Male Bodies [16], which consists of 64 male silhouettes arranged on a 2D grid, with muscularity increasing vertically and body fat increasing horizontally. Similarly, female participants completed the Body Image Matrix of Thinness and Muscularity—Female Bodies [17], which follows the same format with 64 female silhouettes. Athletes were asked to respond to two questions: (1) to identify the figure that best reflects their current body (Perceived Current Body) and (2) to indicate the figure that represents their ideal body (Ideal). Selections were converted to numeric codes according to the procedures established in the original validation studies [16,17]. Additional items assessed whether bodybuilding had positively, negatively, or not at all influenced body-image perception.

### 2.3. Statistical Analysis

No a priori sample size calculation was performed. However, given the final sample size of 60, the study was sufficiently powered (80%, α = 0.05) to detect moderate-to-large between-sex differences (Cohen’s d ≈ 0.65). Therefore, findings that are not statistically significant should be interpreted with caution, especially when the effect size is small.

For continuous variables, descriptive statistics were reported as mean and standard deviation (SD), while categorical variables were reported as frequencies. Variables assessed using 5-point Likert scales were treated as approximately continuous variables for the purpose of statistical analysis. The hypothesis of frequency equality was tested by the Pearson Chi-Squared (χ^2^) test. Data distribution normality was assessed using the Shapiro-Wilk test.

A two-way ANOVA was performed to examine differences between groups across sex and experience, including their interaction (sex × experience). The adequacy of the model was assessed by checking the normality of residuals. The Breusch-Pagan/Cook-Weisberg test was used to assess heteroskedasticity in the variables. For variables with significantly unequal variances, the ANOVA with weighted least squares (WLS) was used, with weights calculated from the residuals to account for heteroscedasticity. Instead, if residuals did not meet the assumption of normality, a non-parametric ANOVA based on ranks was applied. The effect size was evaluated using partial eta squared (η_p_^2^) and classified as: small effect = 0.01, medium effect = 0.06, and large effect = ≥0.14 [36]. When ANOVAs were statistically significant (*p* < 0.05), a Tukey post hoc test was performed to identify specific group differences. For categorical variables, frequency distributions were calculated within each sex × experience subgroup. The significance level was set at *p* < 0.05 for all analyses. Statistical analyses were performed using Stata (Stata/SE 18.0 for Mac).

## 3. Results

Participants were distributed across experience levels as follows: among men, 7 (24.1%) were classified as novice, 12 (41.4%) as intermediate, and 10 (34.5%) as advanced, whereas among women, 13 (41.9%) were classified as novice, 5 (16.2%) as intermediate, and 13 (41.9%) as advanced.

Athletes’ anthropometric characteristics by sex and competitive experience are summarized in Table 1.

Compared to women, men were, as expected, significantly taller and heavier, with higher BMIs and lower minimum competition body weights. Competitive experience had negligible effects on these parameters. Detailed effect size analyses are provided in Supplementary Table S1.

Overall, 91.6% of bodybuilders received coaching; however, coaching supervision did not differ by sex (χ^2^ = 0.30, *p* = 0.586) or experience level (χ^2^ = 0.21, *p* = 0.900).

Regarding who settled the nutrition plan and workout schedule, 95% of bodybuilders were guided by a professional, while only a small minority planned training and diet autonomously. In many cases, the same person prescribed both training and diet, while a smaller proportion reported a multidisciplinary approach involving separate professionals, namely a nutritionist and a trainer. Chi-square analyses showed no significant differences in who sets the nutrition and training plans by sex (χ^2^ = 0.38, *p* = 0.828) or experience level (χ^2^ = 8.77, *p* = 0.067).

A substantial proportion of female athletes reported menstrual alterations during competition preparation, with irregular cycles, cycle suspension, or amenorrhea common across all levels of competitive experience. Overall, 60–77% of athletes reported some form of menstrual disturbance, with amenorrhea more prevalent among less experienced athletes. Intermediate and advanced athletes also reported having faced these issues in the past, which often resolved as their competitive experience increased. Despite these apparent differences in percentage, the Pearson chi-square test indicates that the menstrual disturbance is not dependent on experience (χ^2^ = 4.82, *p* = 0.567).

Moreover, both men and women frequently reported decreased libido and sleep disturbances during competition preparation. Among men, decreased libido was consistently high across experience levels, ranging from 72.3% to 85.7%, while among women, it was particularly elevated in intermediate-level athletes, with 80% reporting reductions in sexual desire.

Sleep disturbances increased with experience in men, from 43% in novices to 70% in advanced athletes. Among women, the highest prevalence was observed in advanced athletes, with 85% reporting sleep alterations.

Overall, preparation negatively impacted both sexual function and sleep in most athletes, with some evidence suggesting that men experienced stronger experience-related increases in sleep disturbances. Chi-square analyses revealed no significant associations between sex or experience level and either libido (sex: χ^2^ = 0.873, *p* = 0.350; experience: χ^2^ = 0.210, *p* = 0.900) or sleep disturbances (sex: χ^2^ = 1.681, *p* = 0.195; experience: χ^2^ = 1.212, *p* = 0.545).

Phase-specific ratings of stress, irritability, hunger, satiety, and insomnia differed clearly across the bulk, preparation, and post-competition phases. Specifically, during the bulk phase, athletes generally reported lower stress and irritability, reduced hunger, and greater satiety. In contrast, the competition preparation phase was characterised by increased hunger and decreased satiety, while stress, irritability, and insomnia rose notably.

Following competition, hunger levels decreased, and feelings of satiety became more variable. However, many athletes continued to experience disturbances to their mood and sleep.

Table 2 presents the ratings of perceived stress, irritability, hunger, satiety, and insomnia across the three phases. A significant main effect of sex was observed for stress, irritability, and insomnia in the post-competition phase, whereas no significant main effects of experience and sex × experience interactions were observed.

Effect size analyses are provided in Table S2.

Body weight was frequently monitored, with daily monitoring the most common frequency, particularly among men, both novice and advanced. Notably, only women reported weighing themselves multiple times per day, especially among those with intermediate experience. The Pearson chi-square test indicates no significant association with sex (χ^2^ = 10.39, *p* = 0.065) and experience (χ^2^ = 10.82, *p* = 0.371). Although women check their weight less frequently on average, they reported that body weight has a greater impact on their mood: across experience subgroups, approximately two-thirds of all women indicated that weight affects their emotional state, while the percentages were lower among men. Consistent with this, chi-square analysis showed a significant association between sex and the impact of body weight on mood (χ^2^ = 8.28, *p* = 0.004).

Binge eating episodes were frequently reported by 72% athletes, particularly after competitions. This was observed in both male and female athletes of all experience levels. Consistent with this, chi-square analysis indicates no significant association with sex (χ^2^ = 1.62, *p* = 0.204) and experience (χ^2^ = 1.16, *p* = 0.559).

In contrast, the use of extreme weight loss strategies, such as diuretics, laxatives, self-induced vomiting, or unplanned compensatory exercise, was relatively rare, suggesting that most athletes relied on dietary and training strategies rather than more overtly pathological methods. No significant associations were observed between sex (χ^2^ = 0.934, *p* = 0.627) or experience (χ^2^ = 6.16, *p* = 0.188) and extreme weight loss behaviours.

Table 3 reports the ORTO-15 questionnaire data, while the full content of the ORTO-15 questionnaire items is provided in Table S3.

Mean total ORTO-15 scores across all sex × experience subgroups were below the conventional cut-off of 40, suggesting a potential risk of orthorexia nervosa. No significant main effects of sex or experience were found for the total score. However, item-level analyses revealed significant differences in specific ORTO-15 items. Specifically, significant main effects of sex were observed for items related to confusion when purchasing food (item 2)**,** the influence of mood on eating habits (item 9), and self-esteem linked to healthy eating (item 10). A significant main effect of experience was observed for the item related to allowing oneself dietary transgressions (item 8) and preoccupation with food thoughts for more than three hours per day (item 7); however, post hoc comparisons revealed less preoccupation for advanced participants (<3 years 95% CI: −1.59; −0.57; 3–5 years 95% CI: −1.61; −0.09). Moreover, significant sex × experience interactions were found for items concerning preoccupation with food thoughts for more than three hours per day (item 7) and perception that supermarkets offer only unhealthy food options (item 14). Post hoc analysis revealed that novice males reported greater preoccupation with food compared to novice females (95% CI: 0.18; 1.22) and advanced females (95% CI: 0.22; 1.52). Conversely, advanced males were less worried than novice females (95% CI: −2.37; −0.22) and males with less experience (<3 years 95% CI: −3.18; −0.81; 3–5 years 95% CI: −2.68; −0.38). Moreover, novice females (95% CI: −36.48; −7.64) and advanced females (95% CI: 3.57; 34.05) had more perceptions of unhealthy food than intermediate females. Additionally, intermediate males reported greater concern about unhealthy food than females with the same level of experience (95% CI: 0.42; 37.28). Effect size analysis is reported in Table S4.

EAI-3 data are summarised in Table 4, while the full wording of the EAI-3 questionnaire items is provided in Table S5.

Overall, EAI-3 total scores did not significantly differ by sex or competitive experience. However, mean total EAI-3 scores were higher than the conventional cut-off of 34 in novice females and advanced males, suggesting a potential risk of exercise addiction in these subgroups.

However, significant effects emerged at the item level. Specifically, both sex and experience, and their interaction, had significant main effects on the use of physical exercise to change mood (item 3), indicating differences between males and females and across experience levels. These findings suggest that both sex and experience influence the tendency to use exercise as a mood regulation strategy, with the effect of sex varying depending on training experience. Post hoc comparison revealed that participants with 3–5 years of experience use exercise to regulate mood more than novice participants (95% CI: 1.44; 13.16). Regarding sex × experience interaction, advanced females with more than 5 years of experience reported using exercise for mood regulation less than novice females (95% CI: −17.71; −0.54), whereas novice males reported lower use compared to all female subgroups (<3 years 95% CI: −26.40; −11.99; 3–5 years 95% CI: −28.57; −4.99; >5 years 95% CI: −19.96; −0.18). Moreover, intermediate males reported greater use than novice males (95% CI: 7.44; 26.59). Feelings of guilt when missing a scheduled workout or when a workout did not go as planned (item 7) were significantly influenced by the sex × experience interaction, suggesting that the impact of sex on workout-related guilt depends on the individual’s training experience. Post hoc analyses showed that intermediate females reported lower feelings of guilt than novice females (95% CI: −5.47; −0.84). Effect size analyses are provided in Table S6.

Body-image descriptive data based on the Body Image Matrices are presented in Table 5, with perceived and ideal body fat and muscle values shown separately.

Females reported higher perceived and ideal body fat than males across all experience levels, whereas males showed higher perceived and ideal muscle values. Overall, there was a general tendency for perceived body fat to be higher than the ideal and for perceived muscle to be lower than the ideal, a pattern also reflected in the fat and muscle FID indices.

A two-way ANOVA conducted on the log-transformed Manhattan distance scores revealed no significant main effect of sex (F_(1, 59)_ = 0.04, *p* = 0.84) and of experience (F_(2, 58)_ = 0.58, *p* = 0.57). However, a significant sex × experience interaction was found (F_(2, 58)_ = 6.47, *p* < 0.01). Post hoc analyses did not reveal significant differences between experience groups within either sex.

**Figure 1:**
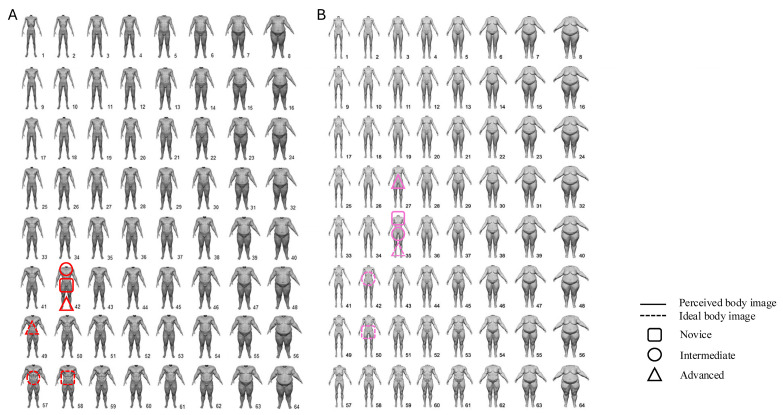
Perceived and ideal body image matrix, separated by sex ((**A**): Males; (**B**): Females) and experience. Adapted from Arkenau et al. (2020) [16] and Steinfeld et al. (2020) [17], licensed under CC-BY 4.0.

Figure 1 shows the body image matrix used in the study, with the average silhouettes selected by participants for perceived and ideal body image, grouped by sex and experience level.

Furthermore, regarding the perceived influence of bodybuilding on body image, the majority of athletes reported a positive influence, while a smaller proportion reported no or a negative influence.

## 4. Discussion

This study aimed to investigate sex- and experience-related differences in eating behaviors, training management, hormone-related symptoms, and self-perception among competitive bodybuilders. Overall, the findings support previous work suggesting that physique sports may represent a context associated with disordered eating behaviors, body image disturbance, and problematic exercise patterns. This is in line with research across different sport disciplines highlighting relationships between aesthetic or weight-sensitive sports and increased body dissatisfaction, restrictive eating behaviors, and exercise dependence [33,37,38,39]. Moreover, this study contributes to the literature by directly comparing male and female athletes across different competitive levels [11,21].

The phase-specific pattern of hunger and satiety reported by subjects is consistent with known physiological adaptations to changes in energy availability. During the bulking phase, characterised by higher caloric intake, athletes reported lower hunger and greater satiety. In contrast, competition preparation, marked by progressive caloric restriction aimed at enhancing muscle definition and reducing fat mass, was associated with increased hunger and decreased satiety. These modifications in appetite regulation are closely associated with alterations in leptin, a hormone secreted by adipose tissue that plays a central role in energy balance [40]. Across the typical phases of bodybuilding, fluctuations in caloric intake are likely to lead to corresponding changes in leptin levels, which, in turn, influence subjective perceptions of hunger and satiety [9]. The symptom profile reported in the present sample, including altered hunger and satiety, increased stress, irritability, and insomnia, reduced libido and menstrual disturbances, closely resembles the endocrine and psychological profile previously described in physique athletes experiencing low energy availability [5,7,8].

Another salient characteristic of the sample was the high level of monitoring of physical appearance, experienced not only as a performance-related behaviour but also as emotionally demanding. Frequent body checking and weight monitoring were perceived as stressful, particularly when changes deviated from expectations. Female athletes, in particular, reported greater concerns about weight and body shape, and more rigid adherence to dietary plans, especially at lower levels of experience. Intense feelings of guilt often accompanied deviations from prescribed dietary protocols. These findings are consistent with previous evidence indicating that female physique athletes experience heightened body shape concerns, especially in the post-competition period [22], and suggest that such concerns may be particularly pronounced in women. The combination of constant body monitoring and rigid dietary adherence may contribute to the development of orthorexic tendencies. Across all subgroups, mean ORTO-15 scores were below 40, the cut-off traditionally interpreted as indicative of risk for orthorexia nervosa [23]. Item-level analysis revealed that novice males reported greater daily preoccupation with food, which decreased with increasing experience, whereas perceptions of unhealthy food differed across experience levels, particularly among female participants. These patterns align with work showing elevated orthorexic traits among bodybuilders and strength athletes compared with control groups [24,25] and with conceptual descriptions of orthorexia as being characterised by rigid dichotomies between so-called “healthy” and “unhealthy” foods and the moralisation of eating [24]. The present findings, therefore, suggest that competitive bodybuilding, irrespective of sex and experience, fosters a high degree of rule-governed eating that may drift into clinically significant orthorexic patterns in a subset of athletes. Despite this dietary rigidity, the majority of athletes reported binge eating episodes, particularly during the post-competition period. This aligns with previous findings in female physique athletes, where increased binge eating has been documented following competition [22], as well as with broader evidence linking extreme dieting, weight cycling, and periods of strict restriction to subsequent loss-of-control eating [1,4,11]. In the present sample, post-competition binges likely reflect the combined effect of prolonged caloric restriction, the elimination of specific foods, and the psychological rebound that occurs once dietary rules are relaxed. These patterns reinforce the notion that contest preparation strategies, if excessively restrictive or prolonged, may increase vulnerability to both orthorexic and binge-type eating within the same individual, depending on the phase of the competitive cycle [1,4].

Problematic exercise-related behaviours were also evident. Although no statistically significant differences by sex or experience emerged in total scores, novice females and advanced males showed a higher risk of exercise addiction, with mean total scores exceeding the cut-off of 34. This suggests that the specific demands of bodybuilding, including high-volume and high-intensity resistance training often combined with substantial cardiovascular exercise, may inherently foster a risk of exercise-related dysfunction [27,28]. Training experience and its interaction with sex influenced emotional and physical responses to exercise. Mood-regulating use of exercise increased with experience in males but decreased in highly experienced females, whereas workout-related guilt was lowest at intermediate experience levels, particularly among females. These patterns are consistent with the construct of vigorexia, also known as muscle dysmorphia, which is characterised by compulsive exercise, distress when training is missed, and conflict with significant others due to training volume [26,30]. Combined with the high importance attributed to muscularity and training, these findings support the view that muscle dysmorphia and exercise addiction represent particularly relevant risks among male bodybuilders, even though female athletes also exhibited elevated scores [21,30].

Regarding body image, clear sex-related differences emerged in both perceived and ideal body composition. Males reported higher perceived and ideal muscle values, aligning with social and sport-specific norms that value large, muscular male bodies [15,16]. In contrast, females reported higher perceived body fat and leaner body ideals, consistent with broader sociocultural standards emphasizing thinness in women [41]. These findings are consistent with competition categories that reward greater muscularity in male divisions and emphasize leanness in female categories. Across groups, bodybuilders generally perceived themselves as having more body fat than their ideal and less muscle than desired, as reflected in the positive FID-fat and negative FID-muscle scores. This pattern suggests a persistent drive toward lower adiposity and greater muscularity, even in a physique-oriented population. Previous studies have typically examined male and female body image separately [20,26,28]. By directly comparing men and women across experience levels, the present study demonstrates that bodybuilding is associated with statistically significant differences in perceived and desired body image between men and women, and that competitive experience substantially reshapes aesthetic ideals among female athletes.

Although perfectionism was not directly assessed in the present study, some of the observed behaviours may be interpreted in light of perfectionistic tendencies. Athletes frequently interpreted any deviation from dietary or training plans as a personal failure, engaged in relentless monitoring of their bodies, and continually pursued an ideal that never fully matched their current physiques. These patterns may reflect tendencies that are consistent with perfectionistic traits. Previous research has linked perfectionism to body dissatisfaction and eating disorders in athletes and non-athletes alike [12]. Within the context of bodybuilding, perfectionism may provide a useful interpretative framework for understanding the behaviours described by participants, including rigid and health-focused eating patterns, intensive training practices, muscle dysmorphia, and post-competition distress when the stage-ready physique cannot be maintained year-round [4,11,21].

This study has several limitations. Its cross-sectional design, the use of a convenience sample, the relatively small and unbalanced sample may limit the generalizability of the findings. In addition, the use of an online self-report questionnaire may have led to self-selection, recall, and response bias. Finally, the lack of objective hormonal and energy availability measures, clinical diagnostic interviews, and detailed information on competitive phase, training frequency, and supplement use should be considered when interpreting the findings.

## 5. Conclusions

In summary, the present findings suggest that competitive bodybuilding is a high-risk sport for the development of disordered eating, orthorexic behaviours, binge eating episodes, muscle dysmorphia, and exercise addiction. They also indicate that these problems manifest differently in men and women and vary with competitive experience. Male athletes appear particularly vulnerable to exercise-related issues associated with the pursuit of extreme muscularity. In contrast, female athletes, especially those with increasing experience, show a stronger shift toward leanness-oriented ideals, more rigid dietary control, and greater concern about changes in body composition.

Across both sexes, prolonged caloric restriction during competition preparation is associated with symptoms suggestive of hormonal dysregulation. Moreover, the combination of strict weight-control practices, body image pressures, and perfectionistic tendencies creates a fertile ground for psychopathology. These findings underscore the need for sport-specific prevention and early-intervention strategies, highlighting the importance of multidisciplinary support and a culture that values health and psychological well-being alongside aesthetic and performance outcomes [4,11,12].

## Figures and Tables

**Table 1:** Anthropometric characteristics of competitive bodybuilders by sex and competitive experience.

	Female						Male						Two-Way Anova					
	<3 y		3–5 y		>5 y		<3 y		3–5 y		>5 y		Sex		Experience		Sex × Experience	
	Mean	SD	Mean	SD	Mean	SD	Mean	SD	Mean	SD	Mean	SD	F_(1, 59)_	*p*	F_(2, 58)_	*p*	F_(2, 58)_	*p*
Stature (cm)	161.5	6.3	162.0	5.9	163.5	7.8	171.7	5.2	175.1	6.4	176.5	4.3	49.76	<0.001	1.49	0.236	0.31	0.738
BM (kg)	55.0	6.01	53.8	5.7	55.1	7.5	77.7	8.6	80.5	10.8	85.0	9.2	133.92	<0.001	1.11	0.338	0.95	0.394
BMI (kg/m^2^)	21.1	1.8	20.5	1.8	20.6	2.2	26.3	2.5	26.2	2.2	27.3	2.4	96.36	<0.001	0.31	0.733	0.56	0.576
mBM (kg)	50.4	4.9	50.0	3.6	49.5	4.8	70.6	8.8	72.5	8.1	74.5	5.3	176.79	<0.001	0.32	0.730	0.76	0.473

Note: BM = Body Mass; BMI = Body Mass Index; mBM = minimum weight during competition.

**Table 2:** Perception of feelings of stress, irritability, hunger, satiety, and insomnia across bulk, preparation, and post-competition phases in bodybuilder athletes by sex and competitive experience.

		Female						Male						Two-Way Anova					
		<3 y		3–5 y		>5 y		<3 y		3–5 y		>5 y		Sex		Experience		Sex × Experience	
Phase		Mean	SD	Mean	SD	Mean	SD	Mean	SD	Mean	SD	Mean	SD	F_(1, 59)_	*p*	F_(2, 58)_	*p*	F_(2, 58)_	*p*
Bulk	Stress	2.1	1.3	2.0	0.0	2.3	1.1	1.7	0.8	2.1	0.8	1.7	0.8	1.25	0.269	0.11	0.900	0.56	0.574
	Irritability	1.8	0.9	1.6	0.6	1.7	0.8	1.6	0.5	1.7	0.8	1.6	0.7	0.13	0.722	0.01	0.989	0.12	0.886
	Hungry	1.5	0.9	2.0	1.0	1.7	1.0	1.6	1.0	1.5	0.9	1.3	0.5	1.37	0.247	0.36	0.696	0.43	0.650
	Satiety	3.5	0.9	2.6	0.9	3.1	1.1	3.7	1.0	3.3	0.8	3.3	1.5	1.48	0.229	1.89	0.160	0.24	0.785
	Insomnia	1.5	0.8	1.2	0.5	1.7	0.9	1.7	1.1	1.7	0.9	1.2	0.4	0.12	0.731	0.21	0.814	1.90	0.159
Preparation	Stress	3.6	1.0	2.8	0.5	3.9	1.1	3.7	0.8	3.4	0.9	3.2	0.9	0.01	0.927	1.54	0.223	2.18	0.123
	Irritability	3.5	1.0	2.6	1.1	3.6	1.3	3.1	1.7	3.5	1.0	3.6	1.0	0.35	0.554	1.05	0.355	1.18	0.316
	Hungry	3.9	1.1	3.2	0.8	4.1	0.9	3.6	1.3	3.5	1.0	3.6	0.7	0.32	0.573	1.13	0.330	0.72	0.493
	Satiety	1.4	0.5	2.0	1.2	1.7	0.9	1.6	0.8	2.2	1.4	1.9	1.1	0.46	0.499	1.47	0.238	0.00	0.998
	Insomnia	2.7	1.3	2.4	1.1	3.0	1.0	2.1	0.9	2.6	1.2	2.2	1.2	1.51	0.223	0.13	0.878	0.81	0.448
Post-Competition	Stress	2.3	1.0	2.6	0.6	2.9	1.4	1.7	0.8	2.0	1.0	1.7	0.8	7.94	0.007	0.49	0.613	0.60	0.550
	Irritability	1.9	0.6	1.8	0.5	2.8	1.4	1.3	0.5	1.8	1.0	1.5	0.5	6.39	0.014	1.80	0.175	2.31	0.108
	Hungry	2.2	1.3	2.6	0.9	3.1	1.3	2.1	1.6	2.3	0.9	2.0	0.9	2.14	0.149	0.47	0.627	1.00	0.373
	Satiety	2.6	1.0	2.4	0.9	2.1	0.9	1.9	0.9	2.8	1.1	2.2	0.9	0.07	0.798	1.14	0.328	1.78	0.178
	Insomnia	1.8	0.8	1.6	0.6	2.2	1.1	1.3	0.5	1.5	0.5	1.3	0.5	6.00	0.017	0.62	0.542	1.38	0.260

**Table 3:** ORTO-15 scores by sex and competitive experience.

	Female						Male						Two-Way Anova					
	<3 y		3–5 y		>5 y		<3 y		3–5 y		>5 y		Sex		Experience		Sex × Experience	
	Mean	SD	Mean	SD	Mean	SD	Mean	SD	Mean	SD	Mean	SD	F_(1, 59)_	*p*	F_(2, 58)_	*p*	F_(2, 58)_	*p*
Item 1	2.8	1.2	2.2	1.1	2.3	0.5	2.7	1.3	3.1	0.7	2.5	0.9	1.85	0.179	0.74	0.480	1.11	0.337
Item 2	1.5	0.9	1.4	0.6	1.5	0.7	1.1	0.4	1.3	0.5	1.3	0.7	4.48	0.039	0.47	0.630	0.23	0.795
Item 3	2.6	0.9	3.0	0.7	3.3	0.6	3.3	0.8	3.3	1.1	3.5	1.0	4.04	0.049	1.79	0.177	0.17	0.843
Item 4	2.5	1.0	3.0	1.0	2.5	0.9	2.7	1.0	3.1	0.9	3.2	1.1	1.57	0.215	0.93	0.402	0.45	0.642
Item 5	2.2	1.0	2.2	0.5	2.2	1.0	1.6	1.0	2.2	0.9	2.1	0.9	0.94	0.336	0.67	0.515	0.43	0.653
Item 6	2.5	1.1	2.4	1.1	2.9	1.3	3.0	1.0	2.7	0.8	2.7	1.2	0.41	0.526	0.28	0.758	0.62	0.541
Item 7	2.9	1.0	3.0	1.0	2.6	1.0	3.6	0.5	3.3	1.1	2.1	1.5	0.08	0.773	13.05	<0.001	9.34	<0.001
Item 8	2.3	0.6	2.8	0.8	2.2	0.8	1.9	0.4	2.4	0.5	2.0	0.8	2.39	0.127	3.53	0.036	0.32	0.726
Item 9	2.7	0.8	2.6	1.1	2.2	0.6	1.6	0.5	2.1	1.0	2.3	1.1	5.01	0.029	0.26	0.773	2.48	0.093
Item 10	2.9	0.8	2.6	0.9	2.6	0.7	3.7	0.8	3.0	0.9	2.8	1.6	4.57	0.037	2.14	0.127	0.37	0.694
Item 11	1.9	0.8	2.2	0.8	2.2	0.8	2.0	0.8	2.3	0.8	1.3	0.7	0.98	0.326	1.77	0.180	3.10	0.053
Item 12	1.2	0.4	1.0	0.0	1.5	0.5	1.7	0.8	1.5	0.5	1.3	0.7	2.72	0.104	0.52	0.598	3.11	0.052
Item 13	2.9	0.9	2.4	0.9	3.0	0.6	2.1	1.1	2.3	1.2	2.1	1.1	3.73	0.058	0.15	0.862	1.00	0.375
Item 14	3.6	0.7	2.8	1.3	3.5	0.7	3.4	0.8	3.7	0.7	3.1	1.3	2.35	0.130	2.31	0.108	11.43	<0.001
Item 15	2.4	0.8	2.6	0.9	3.2	1.0	2.9	0.7	2.3	1.0	2.3	1.1	0.69	0.408	0.35	0.709	2.68	0.077
Total Scores	36.9	2.9	36.2	3.4	37.9	3.1	37.3	3.8	38.4	3.7	34.6	4.0	0.04	0.836	0.52	0.596	3.04	0.055

**Table 4:** Expanded Exercise Addiction Inventory (EAI-3) scores by sex and competitive experience.

	Female						Male						Two-Way Anova					
	<3 y		3–5 y		>5 y		<3 y		3–5 y		>5 y		Sex		Experience		Sex × Experience	
	Mean	SD	Mean	SD	Mean	SD	Mean	SD	Mean	SD	Mean	SD	F_(1, 59)_	*p*	F_(2, 58)_	*p*	F_(2, 58)_	*p*
Item 1	4.3	1.0	4.0	1.4	3.6	1.5	3.4	1.7	4.2	1.3	4.3	1.3	0.00	0.980	0.10	0.906	1.66	0.198
Item 2	3.4	1.9	3.4	0.6	3.1	1.4	3.6	1.6	2.8	1.5	2.5	1.8	0.53	0.469	0.95	0.394	0.35	0.709
Item 3	5.2	0.9	4.6	1.1	4.5	1.0	3.1	1.6	4.3	1.7	3.5	2.1	8.61	0.004	4.97	0.010	9.58	<0.001
Item 4	4.2	2.1	3.2	1.8	4.5	1.3	2.9	1.8	4.3	1.4	3.6	1.7	0.55	0.461	0.50	0.611	2.32	0.107
Item 5	4.9	1.4	4.2	1.3	4.2	1.5	4.6	1.4	3.8	1.5	3.5	2.0	1.30	0.258	1.78	0.177	0.10	0.907
Item 6	3.4	2.0	2.2	1.1	3.2	1.6	2.9	1.2	3.4	1.7	3.1	1.5	0.23	0.636	0.20	0.820	1.20	0.309
Item 7	5.2	1.0	2.0	1.4	4.3	1.4	3.4	1.8	4.1	1.4	3.6	1.9	0.08	0.776	2.94	0.061	6.38	0.002
Item 8	4.3	1.8	3.8	1.5	3.6	1.7	2.7	1.6	3.8	1.6	4.2	1.8	0.59	0.444	0.29	0.749	2.21	0.119
Total Scores	34.8	8.0	27.4	6.8	30.9	7.9	26.6	8.7	30.7	7.8	38.3	9.8	1.23	0.271	0.17	0.845	1.93	0.155

**Table 5:** Perceived and Ideal body-image scores (Body Image Matrix codes), separated by fat and muscle components.

	Female						Male					
	<3 y		3–5 y		>5 y		<3 y		3–5 y		>5 y	
	Mean	SD	Mean	SD	Mean	SD	Mean	SD	Mean	SD	Mean	SD
Perceived body fat	3.3	0.5	3.0	0.7	2.9	0.8	1.7	1.3	2.3	1.6	2.0	1.3
Perceived body muscle	5.2	1.5	5.4	2.5	4.3	2.5	6.3	1.3	6.3	1.6	6.1	1.2
Ideal body fat	2.5	0.7	2.4	0.6	2.5	1.0	1.7	1.3	1.3	0.7	1.1	0.3
Ideal body muscle	7.5	0.5	6.2	3.0	5.2	2.6	7.6	0.8	7.7	0.7	7.1	0.9
FID body fat	0.9	0.8	0.6	0.9	0.3	0.9	0.0	0.0	1.0	1.8	0.9	1.3
FID body muscle	−2.3	1.3	−0.8	0.5	−0.9	1.4	−1.3	1.4	−1.4	1.3	−1.0	0.8
Manhattan distance	3.2	1.7	1.4	0.9	1.5	1.8	1.3	1.4	2.9	1.7	1.9	1.8

## Data Availability

The original contributions presented in this study are included in the article/Supplementary Material. Further inquiries can be directed to the corresponding authors.

## Supplementary Materials

The following supporting information can be downloaded at: https://www.mdpi.com/article/10.3390/jfmk11020236/s1, Table S1: Effect size of anthropometric characteristics by sex and competitive experience; Table S2: Effect size of feelings of stress, irritability, hunger, satiety and insomnia across bulk, preparation and post-competition phases in bodybuilders athletes by sex and competitive experience; Table S3: ORTO-15 Items; Table S4: Effect size of ORTO-15 scores by sex and competitive experience; Table S5: EAI-3 Items; TableS6: Effect size of Expanded Exercise Addiction Inventory (EAI-3) scores by sex and competitive experience.

## Abbreviations

The following abbreviations are used in this manuscript:

RED-S	Relative Energy Deficiency in Sport
EAI-3	Exercise Addiction Inventory
FID	Feel-Ideal Discrepancy

## References

[1] Helms E.R., Aragon A.A., Fitschen P.J. (2014). Evidence-based recommendations for natural bodybuilding contest preparation: Nutrition and supplementation. J. Int. Soc. Sports Nutr..

[2] Escalante G., Stevenson S.W., Barakat C., Aragon A.A., Schoenfeld B.J. (2021). Peak week recommendations for bodybuilders: An evidence-based approach. BMC Sports Sci. Med. Rehabil..

[3] Iraki J., Fitschen P., Espinar S., Helms E. (2019). Nutrition recommendations for bodybuilders in the off-season: A narrative review. Sports.

[4] Helms E.R., Prnjak K., Linardon J. (2019). Towards a sustainable nutrition paradigm in physique sport: A narrative review. Sports.

[5] Coelho A.R., Cardoso G., Brito M.E., Gomes I.N., Cascais M.J. (2021). The Female Athlete Triad/Relative Energy Deficiency in Sports (RED-S). Rev. Bras. Ginecol. Obstet..

[6] Dipla K., Kraemer R.R., Constantini N.W., Hackney A.C. (2021). Relative energy deficiency in sports (RED-S): Elucidation of endocrine changes affecting the health of males and females. Hormones.

[7] Isola V., Hulmi J.J., Mbay T., Kyröläinen H., Häkkinen K., Ahola V., Helms E.R., Ahtiainen J.P. (2025). Changes in hormonal profiles during competition preparation in physique athletes. Eur. J. Appl. Physiol..

[8] Schoenfeld B.J., Androulakis-Korakakis P., Piñero A., Burke R., Coleman M., Mohan A.E., Escalante G., Rukstela A., Campbell B., Helms E. (2023). Alterations in measures of body composition, neuromuscular performance, hormonal levels, physiological adaptations, and psychometric outcomes during preparation for physique competition: A systematic review of case studies. J. Funct. Morphol. Kinesiol..

[9] Obradovic M., Sudar-Milovanovic E., Soskic S., Essack M., Arya S., Stewart A.J., Gojobori T., Isenovic E.R. (2021). Leptin and obesity: Role and clinical implication. Front. Endocrinol..

[10] Dobranowska K., Plińska S., Dobosz A. (2024). Dietary and lifestyle management of functional hypothalamic amenorrhea: A comprehensive review. Nutrients.

[11] Alwan N., Moss S.L., Elliott-Sale K.J., Davies I.G., Enright K. (2019). A narrative review on female physique athletes: The physiological and psychological implications of weight management practices. Int. J. Sport Nutr. Exerc. Metab..

[12] Wells K.R., Jeacocke N.A., Appaneal R., Smith H.D., Vlahovich N., Burke L.M., Hughes D. (2020). The Australian Institute of Sport (AIS) and National Eating Disorders Collaboration (NEDC) position statement on disordered eating in high performance sport. Br. J. Sports Med..

[13] Kling J., Kwakkenbos L., Diedrichs P.C., Rumsey N., Frisén A., Brandão M.P., Silva A.G., Dooley B., Rodgers R.F., Fitzgerald A. (2019). Systematic review of body image measures. Body Image.

[14] Cash T.F., Fleming E.C., Alindogan J., Steadman L., Whitehead A. (2002). Beyond body image as a trait: The development and validation of the Body Image States Scale. Eat. Disord..

[15] Maurin J., Labossière S., Pomerleau-Fontaine L., Boudreault V., Brassard S., Dion J., Durand-Bush N., Parent S., Soulard A. (2024). Personal risk factors and types of sport associated with drive for thinness and drive for muscularity in NextGen athletes. Front. Nutr..

[16] Arkenau R., Vocks S., Taube C.O., Waldorf M., Hartmann A.S. (2020). The Body Image Matrix of Thinness and Muscularity-Male Bodies: Development and validation of a new figure rating scale for body image in men. J. Clin. Psychol..

[17] Steinfeld B., Hartmann A.S., Waldorf M., Vocks S. (2020). Development and initial psychometric evaluation of the Body Image Matrix of Thinness and Muscularity-Female Bodies. J. Eat. Disord..

[18] American Psychiatric Association (2013). Diagnostic and Statistical Manual of Mental Disorders.

[19] Chapa D.A.N., Johnson S.N., Richson B.N., Bjorlie K., Won Y.Q., Nelson S.V., Ayres J., Jun D., Forbush K.T., Christensen K.A. (2022). Eating-disorder psychopathology in female athletes and non-athletes: A meta-analysis. Int. J. Eat. Disord..

[20] Edwards C.D., Aron C.M. (2021). A perfect storm for athletes: Body dysmorphia, problematic exercise, and disordered eating. Strength Cond. J..

[21] Steele I.H., Pope H.G., Kanayama G. (2019). Competitive bodybuilding: Fitness, pathology, or both?. Harv. Rev. Psychiatry.

[22] Mathisen T.F., Sundgot-Borgen J. (2019). Mental health symptoms related to body shape idealization in female fitness physique athletes. Sports.

[23] Donini L.M., Marsili D., Graziani M.P., Imbriale M., Cannella C. (2005). Orthorexia nervosa: Validation of a diagnosis questionnaire. Eat. Weight Disord..

[24] Horovitz O., Argyrides M. (2023). Orthorexia and orthorexia nervosa: A comprehensive examination of prevalence, risk factors, diagnosis, and treatment. Nutrients.

[25] MacPhail D., Oberle C. (2022). Seeing shred: Differences in muscle dysmorphia, orthorexia nervosa, depression, and obsessive-compulsive tendencies among groups of weightlifting athletes. Perform. Enhanc. Health.

[26] De Pascalis P. (2013). Vigoressia, Quando il Fitness Diventa Ossessione.

[27] Granziol U., Griffiths M.D., Zou L., Yang P., Herschel H.K., Junker A., Akimoto T., Stoll O., Alpay M., Aydın Z. (2023). The Expanded Exercise Addiction Inventory (EAI-3): Towards reliable and international screening of exercise-related dysfunction. Int. J. Ment. Health Addict.

[28] Tariq A., Saad A. (2025). When fitness becomes an obsession: A cross-sectional study investigating the risk of exercise addiction among athletes. BMJ Open Sport Exerc. Med..

[29] Rück C., Mataix-Cols D., Feusner J.D., Shavitt R.G., Veale D., Krebs G., de la Cruz L.F. (2024). Body dysmorphic disorder. Nat. Rev. Dis. Primer.

[30] Underwood M., Olivardia R. (2023). “The day you start lifting is the day you become forever small”: Bodybuilders explain muscle dysmorphia. Health.

[31] Devrim A., Bilgic P., Hongu N. (2018). Is there any relationship between body image perception, eating disorders, and muscle dysmorphic disorders in male bodybuilders?. Am. J. Men’s Health.

[32] Cerea S., Bottesi G., Pacelli Q.F., Paoli A., Ghisi M. (2018). Muscle dysmorphia and its associated psychological features in three groups of recreational athletes. Sci. Rep..

[33] Festino E., Papale O., Di Rocco F., De Maio M., Cortis C., Fusco A. (2024). Effect of Physical Activity Behaviors, Team Sports, and Sitting Time on Body Image and Exercise Dependence. Sports.

[34] Chang C.C., Pan M.C., Shu S.T. (2019). Relationship between self-presentation, exercise dependence, and perceived body image. Int. J. Environ. Res. Public Health.

[35] Terry A., Szabo A., Griffiths M.D. (2004). The Exercise Addiction Inventory: A new brief screening tool. Addict. Res. Theory.

[36] Cohen J. (1988). Statistical Power Analysis for the Behavioral Sciences.

[37] Jankauskienė R., Bacevičienė M. (2019). Body Image and Disturbed Eating Attitudes and Behaviors in Sport-Involved Adolescents: The Role of Gender and Sport Characteristics. Nutrients.

[38] Sabiston C.M., Pila E., Vani M., Thøgersen-Ntoumani C. (2019). Body Image, Physical Activity, and Sport: A Scoping Review. Psychol. Sport Exerc..

[39] Maselli M., Gobbi E., Probst M., Carraro A. (2019). Prevalence of Primary and Secondary Exercise Dependence and Its Correlation with Drive for Thinness in Practitioners of Different Sports and Physical Activities. Int. J. Ment. Health Addict..

[40] Kairaitis R., Minderis P., Lukonaitienė I., Mamkus G., Venckūnas T., Kamandulis S. (2025). Dietary, body composition, and blood leptin variations in fit-model female athletes during the pre-competition period. Nutrients.

[41] Zaccagni L., Gualdi-Russo E. (2023). The impact of sports involvement on body image perception and ideals: A systematic review and meta-analysis. Int. J. Environ. Res. Public Health.

