# Supplementary material for: Between Aesthetics and Health: Disordered Eating, Exercise Addiction, and Body Image in Competitive Bodybuilders
Source: J Funct Morphol Kinesiol. 2026 Jun 13;11(2):236. doi: 10.3390/jfmk11020236 (PMC13301592; doi:10.3390/jfmk11020236)
Supplement: Supplementary file 1 [file jfmk-11-00236-s001.zip › jfmk-4350052-supplementary.pdf]

Table S1. Effect size of anthropometric characteristics by sex and competitive experience.

	Sex		Experience		Sex*Experience	
Variable	η_p^2	95% CI	η_p^2	95% CI	η_p^2	95% CI
Stature (m)	0.48	0.28-0.61	0.05	0-0.18	0.01	0-0.09
BM (Kg)	0.71	0.57-0.79	0.04	0-0.15	0.03	0-0.14
BMI (kg/m^2)	0.64	0.47-0.74	0.01	0-0.09	0.02	0-0.11
mBM (Kg)	0.77	0.65-0.83	0.02	0-0.89	0.03	0-0.13

Abbreviations: BM = Body Mass; BMI = Body Mass Index; mBM = minimum weight during competition; ηp2: partial eta square; CI: confidence interval

Table S2. Effect size of feelings of stress, irritability, hunger, satiety and insomnia across bulk, preparation and post-competition phases in bodybuilders athletes by sex and competitive experience.

		Sex		Experience		Sex*Experience	
	Variable	η_p^2	95% CI	η_p^2	95% CI	η_p^2	95% CI
Bulk	Stress	0.02	0-0.14	0.00	0-0.15	0.02	0-0.11
	Irritability	0.00	0-0.08	0.00	0-0	0.00	0-0.05
	Hungry	0.02	0-0.15	0.01	0-0.09	0.02	0-0.10
	Satiety	0.03	0-0.15	0.07	0-0.20	0.01	0-0.08
	Insomnia	0.00	0-0.08	0.01	0-0.07	0.07	0-0.20
Preparation	Stress	0.00	0-0.04	0.05	0-0.18	0.07	0-0.21
	Irritability	0.01	0-0.10	0.04	0-0.15	0.04	0-0.16
	Hungry	0.01	0-0.10	0.04	0-0.16	0.03	0-0.13
	Satiety	0.01	0-0.11	0.05	0-0.18	0.00	0-0
	Insomnia	0.27	0-0.15	0.00	0-0.06	0.03	0-0.14
Post-Competition	Stress	0.13	0.01-0.29	0.02	0-0.11	0.02	0-0.12
	Irritability	0.11	0.00-0.27	0.06	0-0.19	0.08	0-0.22
	Hungry	0.04	0-0.17	0.02	0-0.11	0.04	0-0.15
	Satiety	0.00	0-0.07	0.04	0-0.15	0.06	0-0.19
	Insomnia	0.10	0.00-0.26	0.02	0-0.12	0.05	0-0.17

Abbreviations: η_p^2: partial eta square; CI: confidence interval

Table S3. ORTO-15 Items.

Item	Question
Item 1	When eating, do you pay attention to the calories of the food?
Item 2	When you go in a food shop do you feel confused?
Item 3	In the last 3 months, did the thoughts of food worry you?
Item 4	Are your eating choices conditioned by your worry about your health status?
Item 5	Is the taste of food more important than the quality when you evaluate food?
Item 6	Are you willing to spend more money to have healthier food?
Item 7	Does the thought about food worry you for more than three hours a day?
Item 8	Do you allow yourself any eating transgressions?
Item 9	Do you think your mood affects your eating behaviour?
Item 10	Do you think that the conviction to eat only healthy food increases self-esteem?
Item 11	Do you think that eating healthy food changes your lifestyle (frequency of eating out, friends, …)?
Item 12	Do you think that consuming healthy food may improve your appearance?
Item 13	Do you feel guilty when transgressing?
Item 14	Do you think that on the market there is also unhealthy food?
Item 15	At present, are you alone when having meals?

Table S4. Effect size of ORTO-15 scores by sex and competitive experience.

	Sex		Experience		Sex*Experience	
Variable	η_p^2	95% CI	η_p^2	95% CI	η_p^2	95% CI
Item 1	0.03	0-0.17	0.03	0-0.13	0.04	0-0.15
Item 2	0.08	0-0.23	0.02	0-0.11	0.01	0-0.08
Item 3	0.07	0-0.22	0.06	0-0.19	0.01	0-0.07
Item 4	0.03	0-0.16	0.03	0-0.14	0.01	0-0.10
Item 5	0.02	0-0.13	0.02	0-0.12	0.02	0-0.10
Item 6	0.01	0-0.10	0.01	0-0.08	0.02	0-0.12
Item 7	0.00	0-0.08	0.33	0.12-0.48	0.26	0.07-0.42
Item 8	0.04	0-0.18	0.12	0-0.26	0.01	0-0.09
Item 9	0.08	0-0.24	0.01	0-0.08	0.08	0-0.22
Item 10	0.08	0-0.23	0.07	0-0.21	0.01	0-0.10
Item 11	0.18	0-0.13	0.06	0-0.19	0.10	0-0.25
Item 12	0.05	0-0.19	0.02	0-0.11	0.10	0-0.25
Item 13	0.06	0-0.22	0.01	0-0.06	0.04	0-0.15
Item 14	0.04	0-0.18	0.08	0-0.22	0.30	0.10-0.45
Item 15	0.01	0-0.12	0.01	0-0.09	0.09	0-0.23
Total Scores	0.00	0-0.06	0.02	0-0.11	0.10	0-0.25

Abbreviations: η_p^2: partial eta square; CI: confidence interval

Table S5. EAI-3 Items.

Items	Question
Item 1	Exercise is the most important thing in my life
Item 2	Concerns have arisen between me and my family and/or my partner about the amount of exercise I do
Item 3	I use exercise as a way of changing my mood (e.g., to get a buzz, to escape, etc.)
Item 4	Over time I have increased the amount of exercise I do in a day
Item 5	If I have to miss an exercise session, I feel moody and irritable
Item 6	If I cut down the amount of exercise I do and then start again, I always end up exercising as often as I did before
Item 7	I feel guilty if I miss planned training or if my training does not go as well as planned
Item 8	I am inclined to train when (or before completely recovered from) illness or injury

Table S6. Effect size of Expanded Exercise Addiction Inventory (EAI-3) scores by sex and competitive experience.

	Sex		Experience		Sex*Experience	
Variable	η_p^2	95% CI	η_p^2	95% CI	η_p^2	95% CI
Item 1	0.00	0-0	0.00	0-0.05	0.06	0-0.19
Item 2	0.01	0-0.11	0.03	0-0.14	0.01	0-0.09
Item 3	0.14	0.01-0.31	0.16	0.01-0.31	0.26	0.07-0.42
Item 4	0.01	0-0.11	0.02	0-0.11	0.08	0-0.22
Item 5	0.02	0-0.15	0.06	0-0.19	0.00	0-0.05
Item 6	0.00	0-0.09	0.01	0-0.07	0.04	0-0.16
Item 7	0.00	0-0.07	0.10	0-0.24	0.20	0.03-0.36
Item 8	0.01	0-0.12	0.01	0-0.09	0.08	0-0.21
Item 9	3.19e-07	0-0	0.14	0.00-0.29	0.34	0.13-0.49
Total Scores	0.02	0-0.14	0.01	0-0.07	0.08	0-0.21

Abbreviations: η_p^2: partial eta square; CI: confidence interval
